# Spasticity of the gastrosoleus muscle is related to the development of reduced passive dorsiflexion of the ankle in children with cerebral palsy: A registry analysis of 2,796 examinations in 355 children

**DOI:** 10.3109/17453674.2011.618917

**Published:** 2011-11-25

**Authors:** Gunnar Hägglund, Philippe Wagner

**Affiliations:** ^1^Department of Orthopedics; ^2^Swedish National Competence Center for Musculoskeletal Disorders, Lund University, Lund University Hospital, Lund, Sweden

## Abstract

**Background and purpose:**

Spasticity and muscle contracture are two common manifestations of cerebral palsy (CP). A spastic muscle may inhibit growth in length of the muscle, but the importance of this relationship is not known. In 1994, a register and a healthcare program for children with CP in southern Sweden were initiated. The child's muscle tone according to the Ashworth scale and the ankle range of motion (ROM) is measured annually during the entire growth period. We have used these data to analyze the relationship between spasticity and ROM of the gastrosoleus muscle.

**Patients and methods:**

All measurements in the total population of children with CP aged 0–18 years during the period January 1995 through June 2008 were analyzed. The study was based on 2,796 examinations in 355 children. In the statistical analysis, the effect of muscle tone on ROM was estimated using a random effects model.

**Results:**

The range of dorsiflexion of the ankle joint decreased in the total material by mean 19 (95% CI: 14–24) degrees during the first 18 years of life. There was a statistically significant association between the ROM and the child's level of spasticity during the year preceding the ROM measurement.

**Interpretation:**

Spasticity is related to the development of muscle contracture. In the treatment of children with CP, the spasticity, contracture, and strength of the gastrosoleus muscle must be considered together.

Spasticity has been defined as “a velocity-dependent increase in tonic stretch reflexes (muscle tone) with exaggerated tendon jerks, resulting from hyperexcitability of the stretch reflex, as one component of the upper motor neurone syndrome” (Lance 1980). The spastic subtypes are the most common manifestations of cerebral palsy (CP) (Scholtes et al. 2006).

A spastic muscle will not allow stretch to the same degree as a muscle with normal tone. As a consequence, spasticity may inhibit growth in the length of the muscle, resulting in the development of muscle contracture—measured as a decreasing range of joint motion (Rang et al. 1986). Other manifestations of CP, such as loss of selective muscle control, muscle weakness, or imbalance between agonists and antagonists may also result in the development of muscle contracture.

In 1994, a CP register and a healthcare program, known as CPUP, were initiated for children with CP in southern Sweden (1.3 million inhabitants) (Hägglund et al. 2005a, b). The child's local physiotherapist fills in a recording form 1–2 times a year. The recording form includes measurement of muscle tone of the gastrosoleus muscle according to the modified Ashworth scale (Bohannon and Smith 1987) and the range of motion of the ankle. These data have been used previously to follow the development of spasticity (Hägglund and Wagner 2008) and ROM (Nordmark et al. 2009) over time in children with CP.

In the present study, we analyzed the relationship between spasticity and contracture of the gastrosoleus muscle in children with CP.

## Patients and methods

The CPUP register includes all children with CP who were born after January 1, 1990 and living in the counties of Skåne and Blekinge in southern Sweden (with a total population of about 1.3 million). The number of children with CP in the area corresponds to a prevalence of 2.4–2.6 per 1,000 living births (Nordmark et al. 2001, Westbom et al. 2007).

Our definition of cerebral palsy is that given by Mutch et al. (1992). The diagnosis is made by the child's neuropediatrician. In the program, a continuing standardized follow-up of gross and fine motor function, clinical findings, and treatment is done. The local physiotherapists examine the child and fill in a recording form twice a year until the age of 6 years, then once a year. There are 13 child habilitation units in the area, with about 80 physiotherapists involved in the CPUP program. The recording form includes the subtype of CP, the gross motor function, and muscle tone measured according to the modified Ashworth scale (Bohannon and Smith 1987). Ranges of motion (ROM) in all major joints are measured with a goniometer. The measurements are performed in standardized positions that are described in a manual accompanying the recording form.

The measurements of muscle tone of the gastrosoleus muscle and passive range of dorsiflexion of the ankle joint are done with the hip and knee extended. The CP subtype is classified according to the Surveillance of Cerebral Palsy in Europe network (SCPE) (2000). The gross motor function is classified according to the gross motor function classification system (GMFCS) (Palisano et al. 1997). In children measured before the publication of the GMFCS system in 1997, the child's GMFCS level reported from 1997 was used.

In the present study, we analyzed all measurements of muscle tone in the gastrosoleus muscle and all measurements of ROM in dorsiflexion of the ankle joint in children with CP from 0 to 18 years of age during the period January 1995 through June 2008. Only children who had their first measurement before 4 years of age were included, and only children with a definite diagnosis of CP. As diagnosis is not always possible before 4 years of age, we excluded children who were younger than 4 years in June 2008. For all children, information from both legs was used in the analysis.

Using these criteria, the study was based on 2,796 examinations (5,592 legs) in 355 children. The distribution of CP subtypes, GMFCS levels, and measurements are presented in Tables 1 and 2. 48 children were treated with tendo Achillis or gastrocnemius lengthening. Only measurements up to the date of surgery were included in these children. 90 children were treated with botulinum toxin in the gastrosoleus muscle on 286 occasions. These children were most often examined more than 3 months after treatment, and they were included in the study.

**Table 1.** Distribution of children in relation to CP subtypes based on the Surveillance of Cerebral Palsy in Europe (SCPE) and GMFCS level

CP subtype	GMFCS					Total
	I	II	III	IV	V	
Spastic						
unilateral	95	13	3	2	0	113
bilateral	45	29	31	31	16	152
Dyskinetic	2	2	3	17	19	43
Ataxic	7	9	1	3	0	20
Mixed	7	3	6	4	7	27
Total	156	56	44	57	42	355

**Table 2.** Distribution of measurements in relation to CP subtypes based on the Surveillance of Cerebral Palsy in Europe (SCPE) and GMFCS level

CP subtype	GMFCS					Total
	I	II	III	IV	V	
Spastic						
unilateral	1,476	196	54	20	0	1,746
bilateral	630	522	536	608	240	2,536
Dyskinetic	20	40	42	290	306	698
Ataxic	76	132	16	46	0	270
Mixed	80	36	56	70	100	342
Total	2,282	926	704	1,034	646	5,592

### Statistics

In the statistical analysis, due to the limited amount of data, the GMFCS levels were divided into two groups: I–III and IV–V. The Ashworth levels 1 and 1+ were gathered in one category, corresponding to the original Ashworth scale (Table 3). The effect of muscle tone on ROM was estimated using a random intercepts model. The model is a more flexible way to analyze unbalanced repeated measures data than the more common methods of analysis of variance (ANOVA), and is closely related to ordinary linear regression models (Fitzmaurice et al. 2004). Two random terms were included in the model in order to account for the covariance structure imposed on data by the inclusion of both legs in all children, one for correlation between data from the same individual and one for correlation between data for the same leg. In the statistical model, the effect of muscle tone on ROM was evaluated using the current level of muscle tone, measured simultaneously with ROM, and age effects were estimated using polynomial regression.

**Table 3.** The original Ashworth scale and the number of examinations at each level in the present study

Score	Description of the muscle tone	Number of examinations
0	Normal tone, no increase in tone	1,586
1	Slight increase in tone producing a catch when joint is moved in flexion or extension	2,151
2	More marked increase in tone through most of range, but joint easily moved	1,033
3	Considerable increase in tone, passive movement difficult	747
4	Affected parts rigid in flexion or extension	75

The development of ROM with age, given a constant level of muscle tone, was predicted using the marginal means of the statistical model and plotted on a graph together with corresponding 95% confidence intervals (CIs). Plotted curves therefore correspond to the estimated population mean ROM given a specific age and level of muscle tone.

The overall effect of muscle tone on ROM was evaluated by considering the average difference in ROM between children with normal muscle tone (Ashworth level 0) and children with a specific elevated level of muscle tone, over the entire follow-up period from 0 to 18 years while correcting for age using the random intercepts model. In order to compare the effects of specific levels of muscle tone on ROM between subgroups, we used the fact that non-overlapping 95% confidence intervals indicate a statistically significant difference between estimates at the 5% level (Schenker and Gentleman 2001). STATA software (StataCorp 2005) was used for the statistical analysis.

### Ethics

The study was approved by the Medical Research Ethics Committee at Lund University (LU-443-99).

## Results

**Figure 1.**
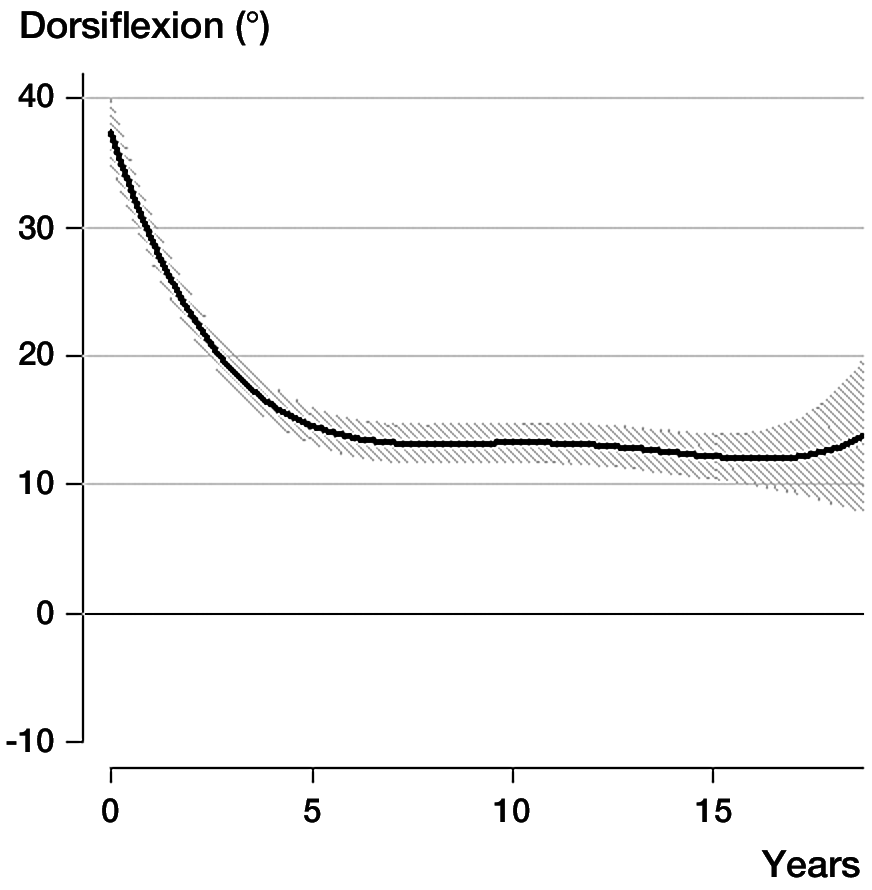
Development of dorsiflexion of the ankle joint (mean, 95% CI) up to 18 years of age in the total sample of 355 children with CP. The analysis is based on 2,796 examinations of both legs in all 355 children.

The observed average decrease in the range of dorsiflexion of the ankle joint in the total material was 19 degrees (95% CI: 14–24) during the period from 0–18 years of age. The observed annual decrease was faster up to approximately 5–6 years of age (Figure 1).

**Figure 2.**
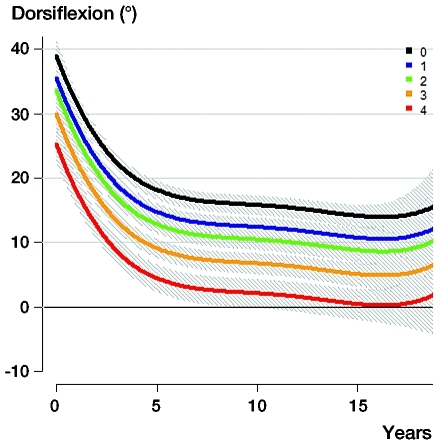
Development of dorsiflexion of the ankle joint (mean, 95% CI) related to level of spasticity according to the Ashworth scale.

The range of dorsiflexion was lower in children with higher levels of spasticity. The predicted development with age for these children is presented in Figure 2. Statistically significant differences in ROM were seen between all levels of spasticity, except between levels 1 and 2.

The decreasing ROM with higher levels of spasticity was seen in all 4 CP subtypes and in both groups of GMFCS levels (Table 4).

**Table 4.** The predicted mean difference in ROM, between children with normal muscle tone and children with different levels of increased muscle tone, over the entire follow-up interval from 0 to 18 years, related to CP subtype and GMFCS level

CP-subtype Ashworth level	GMFCS level	Mean difference	95% CI
Spastic unilateral	I–III		
1		–4.5	(–5.3 to –3.8)
2		–8.1	(–9.4 to –6.8)
3		–15	(–17 to –13)
4		–22	(–30 to –14)
Spastic bilateral	I–III		
1		–1.4	(–2.5 to –0.4)
2		–3.6	(–4.9 to –2.4)
3		–7.6	(–9.1 to –6.2)
4		–17	(–21 to –13)
Spastic bilateral	IV–V		
1		–1.5	(–4.6 to 1.6)
2		–1.2	(–4.0 to 2.2)
3		–6.0	(–9.4 to –2.7)
4		–14	(–20 to –9.0)
Dyskinetic	I–III		
1		–0.3	(–2.4 to 3.0)
2		–2.2	(–6.0 to 1.5)
3		–7.3	(–11 to –3)
4		–29	(–42 to –17)
Dyskinetic	IV–V		
1		–4.8	(–7.7 to –1.8)
2		–7.7	(–11 to – 4.5)
3		–11	(–14 to –7.3)
4		–21	(–28 to –14)
Ataxic **^a^**	I–III		
1		–5.8	(–7.9 to –3.6)
2		–7.1	(–10 to –4.2)
3		–18	(–22 to –14)

**^a^** Only one child with ataxic CP and Ashworth level 4.

The difference in ROM between children with Ashworth level 1 and those with normal muscle tone (Ashworth level 0) varied between CP subtypes and GMFCS groups. The differences ranged from –0.3 (CI: –2.4 to 3.0) degrees in dyskinetic CP and GMFCS I–III to –5.8 (–7.9 to –3.6) degrees in ataxic CP and GMFCS I–III (Table 4). The corresponding differences in ROM for children with Ashworth 4 ranged from –14 (–20 to –9.0) degrees in spastic bilateral CP and GMFCS IV–V to –29 (–42 to –17) degrees in dyskinetic CP and GMFCS I–III. Some of the differences in ROM related to CP subtype and GMFCS levels were statistically significant, indicating an influence of CP subtype and GMFCS level on the effect of spasticity on ROM.

The numbers of examinations in the children with mixed subtype, and in children with spastic unilateral CP and ataxic CP at GMFCS levels IV–V was too small to permit these analyses.

## Discussion

We found that spasticity is related to the development of muscle contracture, measured as reduced ROM. We found a statistically significant difference in ROM across all subgroups between children with normal muscle tone (Ashworth level 0) and those with high muscle tone. The effect of each specific level of muscle tone varied across CP subtypes and GMFCS subgroups. Statistically significant differences were detected for both low and moderate spasticity levels. Differences were also seen for the effect of high spasticity levels, but they lacked statistical significance—possibly due to the relatively low number of measurements available for these children. Furthermore, the apparent increases in ROM at the end of the follow-up period (Figures 1 and 2) are additional statistical artifacts, due to an insufficient number of measurements—which is apparent from the fact that the increases are not statistically significant.

Spasticity is not the only condition responsible for the development of muscle contracture. This is obvious from the fact that even children with normal muscle tone developed reduced ankle ROM. A restricted range of motion caused by reduced selective motor control, dysfunctional co-contraction, muscle weakness, or immobilization could probably also result in muscle contracture.

The classification of CP subtype according to SCPE is made from the most dominant syndrome. Many children with dyskinetic CP also have spasticity (Himmelmann et al. 2007). The ataxic subtype includes children with ataxic diplegia, which explains why some children in this subgroup had increased muscle tone.

We have previously shown that children with spastic CP have increasing muscle tone in the gastrocnemius muscle up to 4 years of age, followed by a decreasing tone up to 12 years of age (Hägglund and Wagner 2008). Both the higher growth rate of the skeleton in the younger children and the higher degree of spasticity at younger ages could explain the faster decrease in ankle ROM before 5 years of age (Figure 1).

If a muscle is shortened, a given change in joint angle will stretch the muscle more than normal. This may potentiate the stretch reflex. In this way, a vicious circle could be set up with spasticity leading to muscle contracture, which would in turn increase spasticity (Nash et al. 1989).

The modified Ashworth scale is the most widely used instrument for assessing spasticity. The modification from the original scale is the addition of one level of measurement (1+) at the lower end of the scale. Some studies have shown problems with interobserver reliability with the Ashworth scale (Hass et al. 1996, Mutlu et al. 2008). The reliability is better with the original scale, as used in the present study (Nuyens et al. 1994, Hass et al. 1996). Some studies have identified the Tardieu scale to be a more sensitive instrument for measuring spasticity (Haugh et al. 2006), but it is to complicated to be used in a surveillance program or in a clinical follow-up of larger samples. In the present analysis, a low reliability—in the absence of systematic errors—would only be evident as underestimation of the effect of muscle tone on ROM. Thus, it will only affect our ability to correctly detect an actual change in ROM with muscle tone.

The validity of the Ashworth scale has been analysed in several studies (Scholtes et al. 2006). Leonard et al. (2001) compared the modified Ashworth scale with myometer measurement of the biceps brachii and found a moderate to high correlation between the methods. Sköld et al. (1998) compared EMG measurement of the quadriceps with estimation of spasticity according to the modified Ashworth scale in adults with spinal cord injury, and found a positive correlation. They concluded that the modified Ashworth scale accurately reflects movement-provoked spasticity.

One limitation of our study was that only the current measurement of muscle tone was used to estimate the effect of spasticity on ROM. The omission of data on past levels of muscle tone from the statistical model was mainly due to lack of balance of measurements of muscle tone and paucity of data due to the fact that younger and older children did not have complete follow-up data, as well as the fact that there were single measurements missing for some individuals. The inclusion of only the current measurement of muscle tone may have caused the past levels to act as confounders, inflating the estimate of the effect of current measurement of muscle tone on ROM. However, since we were interested in measuring the effect of all levels of muscle tone regardless of the time of measurement, this was not an issue for concern.

Our study does not reflect the natural development of spasticity and ROM, as some of the children had been treated to prevent the development of severe contracture. The CPUP program has reduced the number of children with severe contracture and also the number of children who are operated for tendo Achillis lengthening (Hägglund et al 2005b). Children with shortening of the gastrocnemius muscle are often treated with physiotherapy and/or orthoses to reduce the shortening. If the children had not been treated, they would most probably have developed a larger decrease in ROM and the effect of spasticity would have been more pronounced.

Of the 355 children, 90 had been treated with botulinum toxin A in the gastrosoleus muscle on an average of 3 occasions. Most of the treatments were given more than 3 months before the examination. In relation to the 2,796 examinations in the study, these treatments should not have influenced the results. If a child is measured with a temporary lower degree of muscle tone caused by botulinum toxin, this would result in an underestimation of the effect of spasticity on ROM.

Both spasticity and contracture of the gastrosoleus muscle may interfere with ambulation, resulting in toe walking. However, spasticity and contracture may also compensate for weakness of the gastrosoleus muscle. The balance between muscle strength, muscle length, and muscle tone may result in toe walking in the younger child. The degree of spasticity often becomes reduced between 4 and 12 years of age (Hägglund and Wagner 2008). The combination of reduced muscle tone and increased body weight with age might result in a change from toe walking to development of crouch, with increased dorsiflexion of the ankle joint during stance. We therefore recommend delay with decision on tendo Achillis lengthening until it is obvious that a short gastrosoleus muscle is not useful to compensate for the weakness of the muscle.

The association between spasticity and development of contracture and their compensation for weakness is important in the treatment of children with CP. Reducing spasticity or increasing the range of dorsiflexion of the ankle joint is not always beneficial in the long term, and treatment with temporary methods, such as botulinum toxin, intrathecal baclofen pump, serial casting, or orthotics seems safer than permanent methods such as tendo Achillis lengthening or selective dorsal rhizotomy.

In summary, we found that spasticity of the gastrosoleus muscle is related to the development of muscle contracture, measured as reduced passive ankle ROM. Spasticity and contracture of the gastrosoleus muscle may result in toe walking, and may also prevent development of crouch. In treatment planning, spasticity and the contracture and strength of the gastrosoleus muscle must be considered together in a long-term perspective.
